# Temporal Wheat Proteome Remodeling by Deoxynivalenol Reveals Novel Detoxification Signatures and Strategies Across Cultivars

**DOI:** 10.1016/j.mcpro.2025.100988

**Published:** 2025-05-09

**Authors:** Reid Buchanan, Kholoud Shaban, Boyan Liu, Norris Chan, Mitra Serajazari, Jennifer Geddes-McAlister

**Affiliations:** 1 Department of Molecular and Cellular Biology, University of Guelph, Guelph, Ontario, Canada; 2 Department of Plant Agriculture, University of Guelph, Guelph, Ontario, Canada

**Keywords:** deoxynivalenol, detoxification, *Fusarium graminearum*, mycotoxins, proteomics, wheat

## Abstract

Fusarium head blight (FHB) is a globally devastating fungal disease resulting in reduced grain yield and quality, along with contamination of grains with dangerous mycotoxins. Consumption of such mycotoxins through processed food or livestock feed has downstream implications for human and animal health. This interconnectivity across the environment, animal, and human health defines the One Health problem of threatened food safety and security. In this study, we explore remodeling of the wheat proteome upon exposure to a common mycotoxin, deoxynivalenol (DON). We investigate cultivar-specific responses to DON exposure in FHB-susceptible (Norwell) and -resistant (Sumai#3) cultivars across a continuum of exposure (i.e., 24 and 120 h post inoculation) and upon low (i.e., 0.1 mg/ml) and high (1.0 mg/ml) levels of the mycotoxin. This complex experimental design enables us to tease apart the dynamic relationship between each cultivar and DON tolerance. Specifically, we define precise proteins and broad categories of remodeling that are common (i.e., reduction in photosynthesis) and exclusive (i.e., glycosyltransferase) to the cultivars and align with anticipated protective mechanisms. Moreover, we adapted an *in vitro* DON tolerance expression system and determined that induction of an ubiquinol oxidase (UniProt ID: A0A3B6B5K8) provides heightened protection for yeast growth relative to the negative control as well as increased protection compared to a well-defined DON detoxifying protein. Our study suggests a new avenue for the identification and characterization of novel DON detoxifying proteins as putative biomarkers for selected breeding strategies. Such strategies support the production of wheat varieties with increased tolerance to DON for improved global food safety and security.

## Highlights

• Global proteome remodeling during Fusarium head blight in wheat is strongly driven by time and exposure to harmful mycotoxins.
• A reduction in photosynthetic processes and activation of general defense responses upon deoxynivalenol exposure are emphasized within disease susceptible cultivars.
• Specific and protective responses are induced within disease resistant cultivars even upon low level deoxynivalenol exposure.
• Proteomics uncovers novel deoxynivalenol detoxification strategies with potential to improve global food safety and security.

## Graphical Abstract

**Figure:**
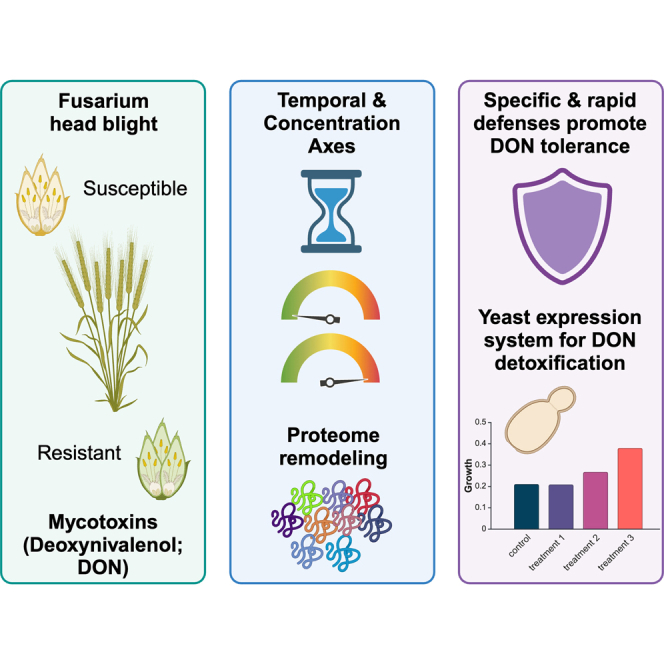

## In Brief

Fusarium head blight (FHB) is a devastating fungal disease that reduces grain yield and quality while contaminating grains with harmful mycotoxins, posing risks to human and animal health. This study examines how the wheat proteome responds to deoxynivalenol (DON) exposure in FHB-resistant and -susceptible cultivars across different time points and toxin levels. We identify key proteins linked to DON tolerance and highlight a novel ubiquinol oxidase that enhances protection, offering new insights for breeding wheat with improved resistance to DON.

Across the globe, cereal crops provide nutritional value through food and feed as well as bioethanol, alcohol, and cosmetics production (1). The breeding of customized cereal cultivars promotes the adaptation of crops to diverse environmental factors impacted by climate change; however, concerns over food safety and security persist (2). Importantly, fungal diseases substantially impact yield loss of cereal crops through growth inhibition or spoilage of grains with disease prevalence rising alongside global temperatures, the emergence of fungicide-resistant strains, and increased food demands for the growing human population (3, 4). Fungal pathogens of cereal crops differ in host range, disease symptoms, and pathogenic mode, with *Fusarium graminearum* threatening a broad host range of cereal crop species to cause Fusarium head blight (FHB) infection (5). Infection of crop material spreads through the presence of the pathogen prior to planting by disruption of fungal spores within the soil through wind and rain, or transmission of the pathogen by insects between plants (6). Typical disease symptoms include the discolouration of spike tissue, mold growth, and seed destruction, which reduce quality and quantity of harvested grains. To withstand the generation of such necrotic host tissue during the infection process, *F. graminearum* shifts from a biotrophic phase to a necrotrophic phase and produces secondary metabolites (e.g., mycotoxins) to facilitate survival and fungal spread to nearby tissues, which further complicates the outcomes of disease (7).

One of the main mycotoxins and virulence factors produced by *F. graminearum* is deoxynivalenol (DON), which confers cytotoxic effects towards the plant (8). Importantly, DON also exerts deleterious effects toward humans upon exposure of DON-contaminated grains or processed foods, and livestock upon consumption of contaminated feed and water run-off (9, 10, 11). DON is a type B trichothecene mycotoxin with toxicity supported by the hydroxyl and acetyl groups on the C3 and C15 positions as well as epoxide at C12 (12, 13). These functional groups allow DON to form hydrogen bonds on the 60S ribosomal subunit, inhibiting protein synthesis at the elongation–termination step of translation, hindering protein production, cellular regulation, and defense responses (14). Moreover, DON contributes to the host production of reactive oxygen species (ROS), which cause further damage to lipids, proteins, and DNA (15, 16). These effects lead to cellular stress and damage, with symptoms in animals and humans, including nausea, vomiting, weight loss, neurological effects, and death (17, 18).

To combat FHB, plants rely on diverse resistance mechanisms (Type I-V), ranging from resisting initial infection by the pathogen, spreading within the spike and kernel, impacting host tolerance to active infection, and reducing DON accumulation (19, 20, 21). Such resistance is associated with the production of ROS, phytohormones (e.g., jasmonic acid and salicylic acid), pathogenesis-related (PR) proteins, and quantitative trait loci (QTLs), which regulate secondary metabolite production as key plant defense responses (22, 23, 24, 25, 26, 27, 28). For example, in *Triticum aestivum* (wheat), the *Fhb1* QTL increases activity of UDP-glycosyltransferase to facilitate glycosylation of the C3 hydroxyl group toward the less toxic DON-3-O-glucoside (D3G), which is sequestered into the plant storage vacuole to prevent further damage and dissemination (29, 30, 31, 32, 33). Additionally, glutathione-*S*-transferase (GST) activity, which is associated with the removal of ROS within a stressed cell, acts upon DON to create a glutathione-S-DON conjugate to reduce DON toxicity through sequestration in the vacuole for storage and processing (34, 35, 36). Despite such plant defense mechanisms against FHB and DON accumulation, knowledge pertaining to the cellular processes of DON detoxification by glutathionation and glycosylation as well as alternative forms of detoxification remain to be fully elucidated.

The interactions between *F. graminearum* and wheat during infection are complex and integrate various components of each biological system, including production of fungal virulence factors and mycotoxins over a time course of infection and fungal growth phases as well as plant defense responses to sequester the pathogen and limit tissue damage (21). Exploring mechanisms of disease tolerance and evasion from both the host and the pathogen perspectives has become feasible over the past 2 decades with increased applications of proteomics technologies (37, 38, 39, 40, 41). To leverage these capabilities, in this study, we comprehensively analyzed protein-level remodeling of *T. aestivum* to 15-ADON across a continuum of time, toxicity, and resistance. We defined growth parameters of FHB-resistant (Sumai#3) and -susceptible (Norwell) wheat varieties at 24 and 120 h post inoculation (hpi) and established this baseline from temporal responses of the wheat varieties upon inoculation with low (0.1 mg/ml) and high (1.0 mg/ml) 15-ADON concentrations. We observed anticipated defense responses of wheat in the presence of 15-ADON (e.g., general defense, PR-proteins, and glutathione metabolism), along with cultivar-specific responses (e.g., glycosyltransferase activation, chitinase production) demonstrating a rapid and elevated proteome response in Sumai#3 compared to Norwell. Prioritization of plant proteins with significantly altered production profiles upon 15-ADON response regardless of cultivar background defined new putative signatures of DON detoxification. Further, our proof-of-concept study demonstrated enhanced tolerance to 15-ADON upon production of an ubiquinol oxidase (UniProt ID: A0A3B6B5K8) within a yeast expression system. Overall, this study defines known and novel mechanisms of wheat proteome remodeling in the presence of 15-ADON and reveals new strategies for mycotoxin detoxification. Additionally, this study provides a foundation to assess cultivar-specific mechanisms of DON detoxification for putative biomarker selection with the long-term goal to inform selected breeding strategies for improved food safety and security.

## Experimental Procedures

### Preparation of DON Inoculum

The 15-acetyl-*O*-DON (Sigma-Aldrich) was dissolved in 0.2% Tween20 (*v/v*) with Milli-Q (18MΩ at 2 5 °C) water solution to concentrations of 1.0 mg/ml and 0.1 mg/ml. The 0.2% Tween20 solution was used as the mock inoculum. Although challenging to connect *in vitro* DON levels with physiologically relevant levels in the field, the range of DON concentration was selected, given cytotoxicity testing of DON (42) and previous analyses (43, 44).

### *T. aestivum* Tissue Cultivation

FHB-resistant (Sumai#3) and -susceptible (Norwell) cultivars of *T. aestivum* were planted in 15-cm pots filled with a mixture of 1:1 nutrient-enriched planting mix (Berger, BM, 6HP) and coarse planting clay (Turface Athletics, MVP, PROFILE Products LLC). The plants were placed in a growth room at 21/18 °C and 16 h light/8 h dark cycles until anthesis (approximately 1–2 months). Upon anthesis, the plants were moved to a growth chamber with a 16 h light/8 h dark cycle set to 27/22 °C, respectively, and relative humidity of 75% to 80%. The samples were separated into the following three inoculation groupings: mock, 0.1 mg/ml 15-ADON, and 1.0 mg/ml 15-ADON with 10 biological replicates per grouping. The samples were point-inoculated with 15 μl of the respective solutions by opening the lemma and palea of the middle two spikes. The inoculated heads were tagged and covered with a plastic bag misted with 2 ml sterile water and harvested, half of each treatment group at 24 h post inoculation (hpi) and the other half at 120 hpi. Immediately after harvesting, the heads were flash-frozen in liquid nitrogen and stored at −80 °C until use.

### Protein Extraction From *T. aestivum* Spike Tissue

Protein extraction from wheat spikes was performed as previously described (45). Briefly, one inoculated spike from each sample was removed from the wheat head and homogenized using steel beads in a tissue homogenizer in the presence of 100 mM Tris-HCl (pH 8.5) with a protease inhibitor cocktail. The samples were transferred into low-protein binding polypropylene tubes, and sodium dodecyl sulphate (SDS) was added (final concentration, 2%) followed by water bath sonication for 1 min. Samples were reduced with 10 mM dithiothreitol and incubated at 95 °C for 10 min at 800 rpm, cooled, and incubated with 55 mM iodoacetamide for 20 min at room temperature in the dark. Samples were centrifuged at 16,200 rpm, and the supernatant was transferred to a new microfuge tube. Acetone was added to a final concentration of 80%, and samples were stored at −20 °C overnight. Samples were centrifuged at 13,500 rpm and 4 °C for 10 min, and the pellet was washed with 80% acetone twice and air dried. The dried pellet was reconstituted in 8 M urea/40 mM HEPES and protein concentration was measured with a bovine serum albumin (BSA) standard (46). Ammonium bicarbonate (50 mM) was added to each sample in a ratio of 3:1, and samples were normalized to 100 μg of protein and digested overnight with LysC/trypsin. Digestion was stopped with 20% acetonitrile/6% trifluoroacetic acid and peptides were desalted using three layers of C18 filters in Stop And Go Extraction (STAGE) tips (47). The purified samples were dried and stored at −20 °C until used for further processing.

### Tandem Mass Tag Labeling

The Tandem Mass Tag (TMT) labels were brought to room temperature and anhydrous acetonitrile was added to a concentration of 25 μg/μl for each label (48). The dried peptides were resuspended in 100 mM triethylammonium bicarbonate (TEAB), and the protein concentration of each sample was measured. The digested protein samples were normalized to set a baseline for the lowest protein concentration across the samples (e.g., 10 μg). Equal protein masses were combined to form an internal standard to run between the labeled tests. The samples were labeled with a ratio of 5 μg label:1 μg peptide. The labeled peptides were combined into their appropriate groupings (15 samples + 1 internal control = 16 TMT channels or reporter ions) with one sample from each label mass per collective sample. The collective samples were dried and stored at −20 °C until mass spectrometry analysis.

### Mass Spectrometry

The purified and labeled peptides were resuspended in a solution of 2% acetonitrile, 0.1% trifluoroacetic acid, and 0.5% acetic acid, separated in-line on a 75-μm by 50-cm PepMap RSLC EASY-Spray column filled with 2-μm C_18_ reverse-phase silica beads (Thermo Fisher Scientific) using an Easy-nLC 1200 high-performance liquid chromatography device (Thermo Fisher Scientific). Peptides were separated along a linear gradient of 3% to 20% buffer B (80% acetonitrile, 0.5% acetic acid) over a 3-h gradient, followed by a wash with 100% buffer B with a 250-nL/min flow rate and analyzed on an Orbitrap Exploris 240 hybrid quadrupole-orbitrap mass spectrometer (Thermo Fisher Scientific). The mass spectrometer was operated in data-dependent mode, switched between one full scan and MS/MS scans of abundant peaks with full scans (*m*/*z* 400–2000) acquired with a resolution of 120,000 at 200 *m*/*z*.

### Raw Mass Spectrometry Data Processing

The mass spectrometry .RAW files were analyzed using MaxQuant software (version 1.6.17.0) (49). The peak list was searched using Andromeda against the reference *T. aestivum* proteome (103,673 sequences, Sept. 15, 2022) from UniProt (50, 51). A list of 16-plex TMT-label spectra shifts was imported into MaxQuant. The following parameters were set: reporter ion MS2, add 16-plex TMT isobaric labels, trypsin enzyme specificity with a maximum of two missed cleavages, and a minimum peptide length of seven amino acids. The “fixed modification” of carbamidomethylation of cysteines and “variable modifications” of oxidation of methionine and N-acetylation of proteins were set. Spectral matching of the peptides was performed with a false discovery rate (FDR) of 1% identified proteins with a minimum of two peptides for protein identification. The mass tolerance for precursor ions was set to 4.5 ppm, and the mass tolerance for fragment ions was set to 20 ppm. All proteins identified and the corresponding information are provided (supplemental Table S1).

### Bioinformatics of Mass Spectrometry Data

The “proteingroups.txt” output file from MaxQuant was analyzed with Perseus (version 1.6.2.2) (52) and visualized with ProteoPlotter (REF) (53). Corrected reporter intensities of all samples were loaded into the matrix for processing. The data were filtered to exclude peptides that were identified as contaminants, reverse peptides, and peptides only identified by site. The readings from the internal controls were removed and the intensities were log_2_ transformed. A file identifying treatment groups was uploaded, and valid value filtering for proteins identified within seven out of 10 replicates was performed. Missing values were replaced with a downshift of 1.8 and a width of 0.3 standard deviations. The data were visualized using principal component analysis (PCA) and proteins with significant changes in abundance between the respective comparisons were identified using volcano plots (Student’s *t* test, *p*-value <0.05; FDR = 0.01, S_0_ = 1). Proteins were sorted by Gene Ontology (GO) protein names and Gene Ontology Biological Processes (GOBP) terms. Proteins with roles related to potential FHB-resistance methods were exported to GraphPad Prism v9 along with the relative intensity values for each associated protein among treatment groups. The values were plotted on Tukey plots and one-way analysis of variance (ANOVA) was performed to identify significance between the treatment groups. Significance was only shown for treatment groups within the same inoculation time setpoints.

### Experimental Design and Statistical Rationale

For this study, 10 biological replicates per treatment, time point, and cultivar were processed for mass spectrometry. The selection of biological samples was designed to ensure adequate representation of FHB-resistance or -susceptibility by cultivar, temporal differences (e.g., 24 and 120- hpi), and treatment response (e.g., 0.1 mg/ml and 1.0 mg/ml DON) as well as untreated controls. The number of biological replicates was determined based on experimental feasibility and power analysis, which determined that a minimum of eight biological replicates should be included in the study to ensure sufficient statistical power and minimize variability due to biological heterogeneity. To ensure statistical significance, we applied a Student’s *t* test with a false discovery rate correction of 5% using the Benjamini-Hochberg method.

### Synthetic Gene Cloning

Genes encoding putative proteins with potential roles in DON detoxification UDP-glucosyltransferase (UGT-6) (33) and ubiquinol oxidase (A0A3B6B5K8, this study) were codon optimized and fused to a FLAG tag encoding sequence before the stop codon. The codon-optimized genes were synthesized by Twist BioScience. They were cloned using the pVUII restriction site in the pYES2 yeast expression vector under the control of the GAL1 inducible promoter using Gibson Assembly. Successful cloning of the genes was confirmed by colony PCR and followed by full vector sequencing by Plasmidsaurus (https://www.plasmidsaurus.com).

### Yeast Transformation With Recombinant Plasmids

pYES2 plasmids encoding candidate proteins of UGT-6 and ubiquinol oxidase (i.e., pYES2-UGT6 and pYES2-Oxidase), pYES2-β-tubulin vector as a negative control along with the pYES2 empty vector were all transformed into W303 *Saccharomyces cerevisiae* yeast strain. Briefly, the W303 strain was grown on Yeast Extract Peptone Dextrose YPD (1% yeast extract, 2% tryptone, 2% glucose) at 30 °C while shaking at 180 rpm until 0.8 to 1.2 OD_600nm_ was reached. Cells were harvested by centrifugation followed by washing with sterile water and 1 M sorbitol. Cells were then resuspended in 1 M sorbitol, and recombinant plasmids were transformed using electroporation (Eporator, Eppendorf).

### Confirmation of UGT-6 and Ubiquinol Oxidase Production in *S. cerevisiae*

Recombinant yeast strains with pYES2 empty vector, pYES2-β-tubulin, pYES2-UGT-6, and pYES2-Oxidase were grown in Synthetic complete media lacking uracil (SC-URA^-^) supplemented with 2% glucose and 1% raffinose overnight at 30 °C while shaking at 180 rpm. Cells were then subcultured into SC-URA^-^ media supplemented with 2% galactose and 1% raffinose and incubated for 4 h to induce protein production. Cells were harvested and were suspended in a lysis buffer (5% SDS, 8 M Urea, 40 mM Tris-HCl pH 6.8, 1 mM EDTA, and bromophenol blue) followed by vortexing for 15 min in the presence of glass beads. Proteins were then separated by SDS-PAGE followed by Western immunoblotting using anti-FLAG antibody (F3165-1MG, Sigma) to detect target proteins.

### DON Resistance Assay and Colony Forming Unit Counting

The DON resistance assay was adapted from previously established yeast growth-deficiency assays (22, 54, 55). Three colonies from each yeast strain producing the proteins of interest (i.e., empty vector, β-tubulin, UGT-6, ubiquinol oxidase) were grown overnight in SC-URA^−^ media supplemented with 2% glucose and 1% raffinose at 30 °C with shaking. Cells were subcultured into SC-URA^−^ media supplemented with 2% galactose and 1% raffinose and incubated for 4 h to induce protein production. The cultures were diluted to reach 0.1 OD_600nm_ in a 96-well plate and exposed to 100 mg/L 15-ADON mycotoxin (Sigma Aldrich). Cells were grown in the absence of 15-ADON as controls. Plates were incubated at 30 °C in a high-speed shaking incubator (900 rpm shaking) for 72 h. The OD_600nm_ values were recorded every 24 h using a Biotek Synergy H1 Hybrid Microplate Reader. Colony-forming unit (CFU) counts were performed every 24 h with 10 μl of cultures serially diluted in SC-URA^−^ media supplemented with 2% galactose and 1% raffinose and incubated on SC-URA^−^ plates at 30 °C for 2 days.

## Results

### Global Proteome Remodeling is Strongly Driven by Time and DON Levels

**Fig. 1:**
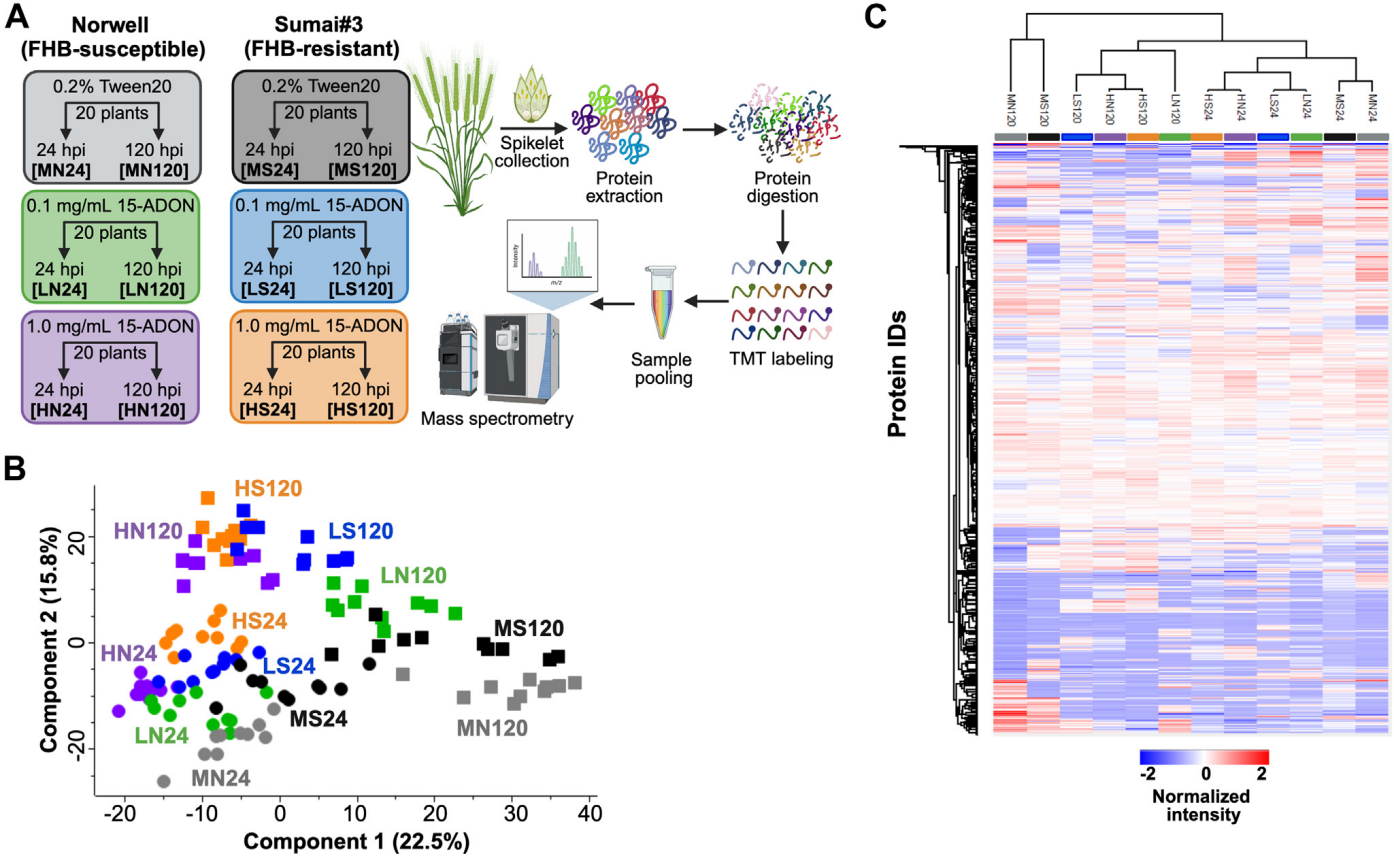
**Global proteome mapping of wheat under exposure to DON.***A*, workflow of wheat cultivars with FHB-resistance (Sumai#3) and -susceptibility (Norwell) across time points (i.e., 24 and 120 hpi) and DON concentrations (i.e., 0.1 mg/ml, 1.0 mg/ml). A single inoculated spikelet was collected, processed, and measured following tandem mass tag labeling for multiplexing. *B*, principal component analysis of *T. aestivum*. *C*, hierarchical clustering by Euclidean distance of protein IDs across averaged same sets (10 biological replicates per treatment). HN, high DON Norwell; HS, high DON Sumai#3; LN, low DON Norwell; LS, low DON Sumai#3; MN, Mock Norwell; MS, Mock Sumai#3.

Based on the devastating impact of DON on cereal crops, we explored responses of the wheat proteome in the presence of low (0.1 mg/ml) and high (1.0 mg/ml) 15-ADON across FHB-resistant (Sumai#3) and -susceptible (Norwell) cultivars (Fig. 1*A*). Our approach aimed to define genotypic regulators of DON accumulation, along with putative temporal differences (24 and 120 hpi) between the cultivars that may provide additional protection towards DON. Our total proteome profiling across the conditions, cultivars, and time points, detected and quantified 5911 unique proteins (5880 proteins after filtering). A principal component analysis (PCA) defined separation of the samples temporally by component 1 (22.5%) and by inoculum (i.e., mock, 0.1 mg/ml 15-ADON, 1.0 mg/ml 15-ADON) by component 2 (15.8%) (Fig. 1*B*). Given intrinsic heterogeneity in plants, these distinct separations by time and treatment underscore the robust quality of our analysis in detecting proteome differences associated with 15-ADON exposure. A global assessment of Euclidean distance by protein IDs across averaged samples demonstrated clustering by time (i.e., 24 hpi vs. 120 hpi) and further clustering by treatment (i.e., mock, low DON, high DON) except by 120 hpi when treatment and cultivar was less easily distinguished (Fig. 1*C*).

### The FHB-Susceptible Cultivar, Norwell, Reduces Photosynthetic Processes and Activates General Defense Responses upon DON Exposure

**Fig. 2:**
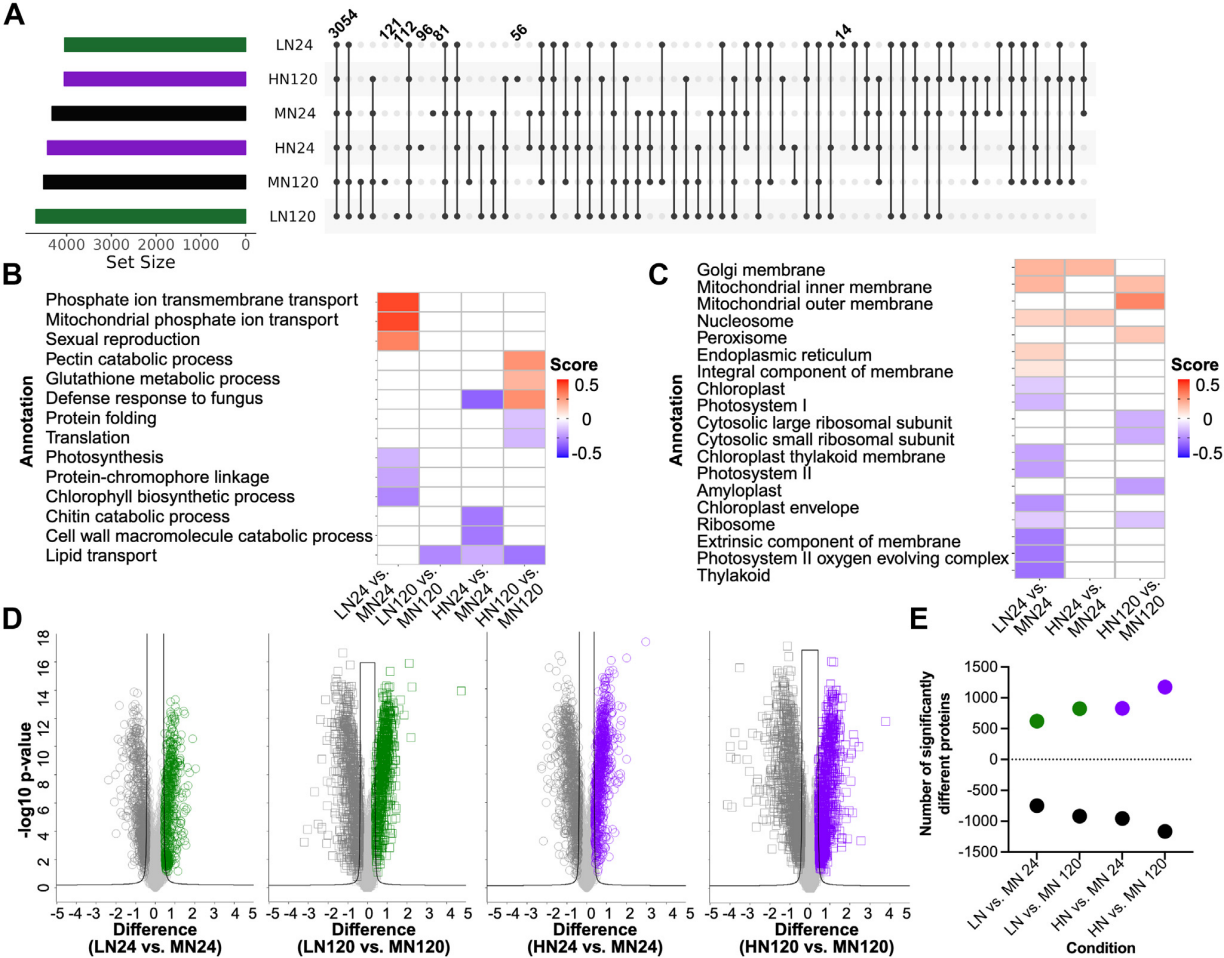
**Proteome remodeling of FHB-susceptible cultivar, Norwell.***A*, upset plot of core (common) and unique (exclusive) proteomes across sample sets. Numbers are indicative of number of proteins defined within the respective category, the core and exclusive proteomes are labeled. *B*, 1D annotation heat map by Gene Ontology Biological Processes. *C*, 1D annotation heat map by Gene Ontology Cellular Component. Student’s *t* test *p*-value <0.05; false discovery rate (FDR) = 5%, <−0.5 score >0.5. *D*, Volcano plots comparing protein abundance profiles between the specific conditions. Student’s *t* test *p*-value <0.05; FDR = 1%, S_0_ = 1. *E*, number of significantly different proteins defined in the volcano plots. Positive numbers represent the number of proteins significantly higher within the treated sample. Negative numbers represent the number of proteins significantly higher in the mock sample. HN, high DON Norwell; HS, high DON Sumai#3; LN, low DON Norwell; LS, low DON Sumai#3; MN, Mock Norwell; MS, Mock Sumai#3.

To explore the impact of DON on the FHB-susceptible cultivar, Norwell, we assessed proteome changes specific to the cultivar over time and at low and high concentrations of 15-ADON. A mock condition was used as a control for treatment and growth differences over time. We defined a core proteome of 3054 proteins across all Norwell conditions with 81 and 121 proteins exclusive to the mock samples at 24 and 120 hpi, respectively, 14 and 112 proteins exclusive to the low DON samples at 24 and 120 hpi, respectively, and 96 and 56 proteins exclusive to the high DON samples at 24 and 120 hpi, respectively (Fig. 2*A*). Next, we performed a 1D annotation enrichment, which evaluates for changes in protein abundance within a defined category of proteins relative to the normal distribution (56). Here, we observed an enrichment of proteins based on Gene Ontology Biological Processes (GOBP) associated with phosphate ion transmembrane transport, mitochondrial phosphate ion transport, and sexual reproduction under low 15-ADON at 24 hpi relative to the mock treatment and a reduction in photosynthesis, protein-chromophore linkage, and chlorophyll biosynthetic processes (Fig. 2*B*). We observed a reduction in chitin catabolic process, cell wall catabolic process, and lipid transport under high 15-ADON at both time points, and protein folding and translation under high 15-ADON. Conversely, we observed an enrichment of proteins involved in pectin catabolic process, glutathione metabolic process, and defense response to fungus under high 15-ADON at 120 hpi. Correspondingly, we observed an enrichment of proteins based on Gene Ontology Cellular Component (GOCC) associated with the mitochondrial inner and outer membranes at low and high 15-ADON, and a reduction of photosynthesis-associated proteins within the chloroplast, photosystem, and thylakoid by 24 hpi under low 15-ADON (Fig. 2*C*). Notably, there was no enrichment of proteins by GOCC in Norwell under low 15-ADON at 120 hpi and the respective mock comparison. Evaluation of proteins with significant changes in abundance between the treated and mock samples defined a mass remodeling of the proteomes (Fig. 2*D*). Moreover, quantification proteins with significantly altered abundance profiles increased over time (i.e., 620 proteins significantly higher in abundance under low 15-ADON at 24 hpi compared to 824 proteins significantly higher in abundance under low 15-ADON at 120 hpi) and upon treatment (i.e., 824 proteins significantly higher in abundance under low 15-ADON compared to 1174 proteins significantly higher in abundance under high 15-ADON at 120 hpi) (Fig. 2*E*). Together, these findings demonstrate substantial remodeling of the Norwell proteome in response to DON treatment at early (i.e., 24 hpi) and late (i.e., 120 hpi) points of exposure leading to general defense responses, reduced photosynthesis, and delayed protective mechanisms (e.g., glutathione metabolism).

### The FHB-Resistant Cultivar, Sumai#3, Induces Specific and Protective Mechanisms Against DON at Low Exposure Levels

**Fig. 3:**
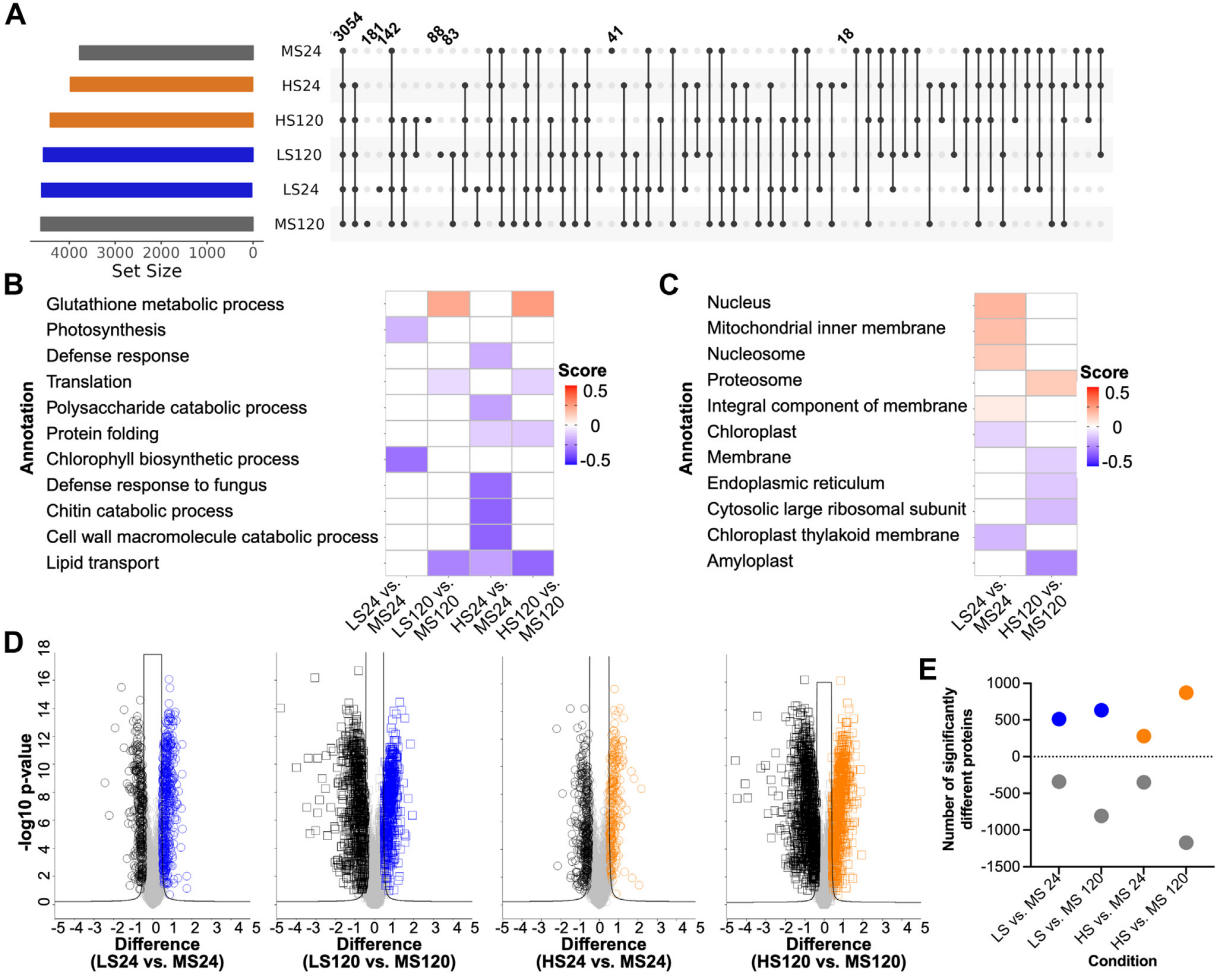
**Proteome remodeling of FHB-resistant cultivar, Sumai#3.***A*, upset plot of core (common) and unique (exclusive) proteomes across sample sets. Numbers are indicative of number of proteins defined within the respective category, the core and exclusive proteomes are labeled. *B*, 1D annotation heat map by Gene Ontology Biological Processes. *C*, 1D annotation heat map by Gene Ontology Cellular Component. Student’s *t* test *p*-value <0.05; false discovery rate (FDR) = 5%, <−0.5 score >0.5. *D*, Volcano plots comparing protein abundance profiles between the specific conditions. Student’s *t* test *p*-value <0.05; FDR = 1%, S_0_ = 1. *E*, number of significantly different proteins defined in the volcano plots. Positive numbers represent the number of proteins significantly higher within the treated sample. Negative numbers represent the number of proteins significantly higher in the mock sample. HN, high DON Norwell; HS, high DON Sumai#3; LN, low DON Norwell; LS, low DON Sumai#3; MN, Mock Norwell; MS, Mock Sumai#3.

Given our findings in Norwell in response to DON, we aimed to evaluate proteome changes in Sumai#3 and identify potential important components of a mycotoxin response that correlate with known FHB resistance of the cultivar. Here, we observed similar proteome coverage with a core proteome of 3054 proteins across all Sumai#3 samples with 41 and 181 proteins exclusive to the mock treatments at 24 and 120 hpi, respectively, 142 and 83 proteins exclusive to low 15-ADON at 24 and 120 hpi, respectively, and 18 and 88 proteins exclusive to high 15-ADON at 24 and 120 hpi, respectively (Fig. 3*A*). An assessment of protein enrichment across GOBP categories showed proteins associated with glutathione metabolic process at 120 hpi under low and high 15-ADON conditions (Fig. 3*B*). We also observed a suppression of proteins associated with photosynthesis and chlorophyll biosynthetic process at 24 hpi under low 15-ADON as well as a reduction of defense response, protein folding, translation, chitin catabolic process, cell wall catabolic process, and lipid transport under high 15-ADON. Correspondingly, by GOCC, we observed an enrichment of proteins associated with the nucleus, nucleosome, and membrane under low and high 15-ADON, and suppression of proteins associated with the chloroplast (Fig. 3*C*). Notably, no enrichment by GOCC was observed under low 15-ADON at 120 hpi or high 15-ADON at 24 hpi. Next, we quantified significant differences in protein abundance profiles across DON treatments compared to mock treatments at early and late time points (Fig. 3*D*). Here, we observed an increase in proteome remodeling over time (i.e., 512 proteins significantly higher in abundance under low 15-ADON at 24 hpi compared to 633 proteins significantly higher in abundance at 120 hpi) with a lower change in protein abundance under high 15-ADON at the early time point (i.e., 512 proteins significantly higher in abundance under low 15-ADON at 24 hpi compared to 280 proteins significantly higher in abundance under high 15-ADON at 24 hpi). Together, these findings demonstrate that the FHB-resistant cultivar, Sumai#3, induces a protective response to DON through the production of glutathione metabolism under low and high 15-ADON exposure. Moreover, similar to Norwell, a reduction in photosynthesis is evident across both cultivars associated with DON exposure, whereas a smaller number of proteins with significant differences in production in Sumai#3 compared to Nowell indicate that the defense responses in the resistant cultivar are more specific and less broad acting.

### Induction of Known Mycotoxin Detoxification Strategies in Sumai#3 may Support Improved Tolerance to DON

**Fig. 4:**
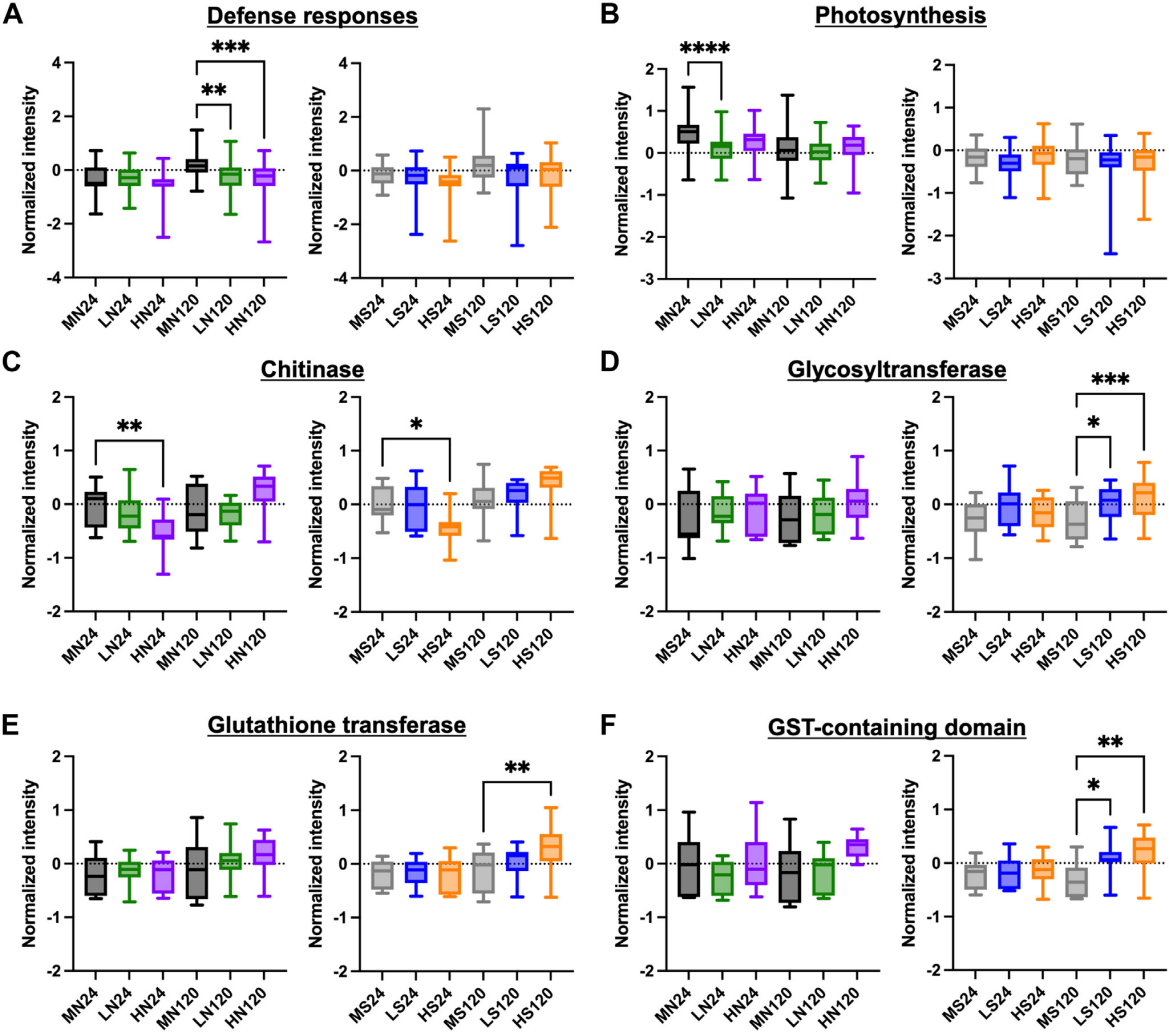
**Comparison of selected protein categories within each sample by normalized intensities.***A*, proteins associated with general defense responses by GOBP (n = 54). *B*, proteins associated with photosynthesis by GOBP (n = 68). *C*, proteins identified as chitinases (n = 15). *D*, proteins identified as glycosyltransferases (n = 25). *E*, proteins identified as glutathione transferases (n = 19). *F*, proteins identified as GST-domain containing proteins (n = 11). Box and whisker plot (standard error) after performing a one-way ANOVA. Statistical significance was identified using a Tukey’s range test, *p*-values <0.05 are shown as ∗, <0.01 as ∗∗, <0.001 as ∗∗∗, and <0.0001 as ∗∗∗∗. HN, high DON Norwell; HS, high DON Sumai#3; LN, low DON Norwell; LS, low DON Sumai#3; MN, Mock Norwell; MS, Mock Sumai#3.

To properly assess protein-level changes associated with DON exposure and tease apart the roles of each cultivar and time point, we performed direct comparisons within each cultivar, and we removed growth-associated proteins from further analysis. Through this approach, we defined important features of each cultivar that may influence plant response to DON. For instance, we observed that the FHB-susceptible cultivar, Norwell, shows a significant reduction in production of defense response proteins at 120 hpi under low and high 15-ADON, whereas no difference is observed in Sumai#3 (Fig. 4*A*). Similarly, Norwell demonstrates a significant reduction in production of proteins involved in photosynthesis by 24 hpi under low 15-ADON conditions (Fig. 4*B*), and for both Norwell and Sumai#3, a significant reduction in chitinases was observed at 24 hpi under high 15-ADON (Fig. 4*C*). Next, given the role of GSTs to reduce ROS within a stressed cell and create a glutathione-S-DON conjugate to reduce DON toxicity (28, 34, 35, 57), we focused on abundance differences of proteins involved in DON detoxification. Specifically, we observed a significant increase in the production of glycosyltransferases in Sumai#3 under low and high 15-ADON exposure at 120 hpi compared to no changes at the early time point or within Norwell (Fig. 4*D*). We found a similar response of glutathione transferases with significantly increased production at 120 hpi under high 15-ADON (Fig. 4*E*), and GST-containing domain proteins with significantly higher abundance at 120 hpi upon low and high 15-ADON exposure (Fig. 4*F*). Given the connection between DON exposure and programmed cell death, and our observation of enrichment within mock samples for proteins associated with protein translation based on GOBP, we explored the putative impact of DON exposure on programmed cell death at the protein level (26, 58). Here, we identified an uncharacterized protein (A0A3B6NNF5) defined by GOBP as a regulator of programmed cell death, as well as a MAP kinase (A0A3B5ZTL2), a regulator of hypersensitive response (59), within the wheat proteome and we observed an increased in production of both these proteins within Norwell (FHB-susceptible cultivar) at 24 hpi under low DON conditions, which maintained abundance throughout the time course of infection and upon higher DON exposure (supplemental Fig. S1). While Sumai#3 showed increased production of such programmed cell death-associated proteins under high DON or at the later (i.e., 120 hpi) time point, suggesting reduced levels of cell death within the FHB-resistant cultivar. Together, these findings demonstrate important changes within families of proteins that may influence the overall defense responses of the cultivars as well as the activation of known DON detoxification proteins within the FHB-resistant cultivar at the later time point.

### Proteome Remodeling Reveals Novel DON Detoxification Strategies in Wheat

**Fig. 5:**
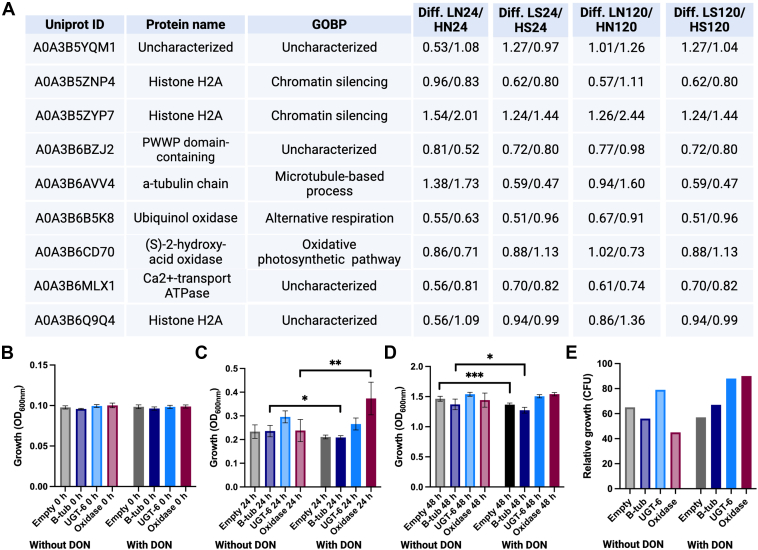
***In vitro* characterization of putative novel proteins associated with DON detoxification.***A*, proteins with common identification as significantly different relative to the mock controls across cultivars, time points, and DON concentrations. Difference indicates normalized intensity fold change between DON treatments and cultivars at the respective time points. *B*, growth by OD_600nm_ of *S. cerevisiae* using the *in vitro* expression assay in the presence of 100 mg/L 15-ADON at 0 hpi. *C*, growth by OD_600nm_ of *S. cerevisiae* using the *in vitro* expression assay in the presence of 100 mg/L 15-ADON at 24 hpi. *D*, growth by OD_600nm_ of *S. cerevisiae* using the *in vitro* expression assay in the presence of 100 mg/L 15-ADON at 48 hpi. Student’s *t* test *p*-value <0.05∗; *p*-value <0.005∗∗; *p*-value <0.001∗∗∗. *E*, relative growth by colony forming unit counts of *S. cerevisiae* using the *in vitro* expression assay in the presence of 100 mg/L 15-ADON at 24 hpi. Filled bar = with DON; shaded bar = without DON. HN, high DON Norwell; HS, high DON Sumai#3; LN, low DON Norwell; LS, low DON Sumai#3; MN, Mock Norwell; MS, Mock Sumai#3.

Given our observations of substantial proteome remodeling for Norwell and Sumai#3 upon exposure to DON, we aimed to identify specific wheat proteins with putative novel roles in DON detoxification or tolerance within the cultivars. Our assessment focused on proteins demonstrating a significant increase in abundance in both Sumai#3 and Norwell upon exposure to DON independent of mycotoxin concentration and time. Here, we identified nine common proteins and explored their putative roles against DON through GOBP annotations (Fig. 5*A*). We observed two oxidases, including ubiquinol oxidase and (S)-2-hydroxy-acid oxidase, along with three histone H2As, an alpha-tubulin chain protein, a calcium-transporting ATPase, and two uncharacterized proteins. Given the previous observation of increased transcriptional activation of oxidases in the presence of 15-ADON in *Escherichia coli* (60) and its homolog AOX1 up-regulated in wheat in response to DON (61) and the role of oxidases in the enzymatic treatment of mycotoxins (62), we tested the corresponding gene encoding for ubiquinol oxidase (UniProt ID: A0A3B6B5K8), along with positive (i.e., UDP-glycosyltransferase, UGT6) (33) and negative (i.e., β-tubulin) controls for DON detoxification. The genes were cloned and produced within a *S. cerevisiae* expression system and survival of the yeast in the presence of 15-ADON was determined (55). At 0 hpi in the presence or absence of 15-ADON, we did not observe any significant differences in yeast cell growth upon production of the indicated proteins (Fig. 5*B*). At 24 hpi, we observed a significant reduction in yeast cell growth during ß-tubulin production in the presence of 15-ADON compared to the absence of 15-ADON, and a significant increase in yeast cell growth during ubiquinol oxidase production in the presence of 15-ADON compared to the absence of 15-ADON (Fig. 5*C*). These data suggest increased tolerance of the yeast strains producing ubiquinol oxidase in the presence of 15-ADON, along with an ability to not only survive in the presence of DON but also grow, indicating complex mechanisms underscoring ubiquinol oxidase’s functional roles in DON detoxification. We observed a consistent level of yeast growth upon production of UGT-6 in the presence or absence of 15-ADON, supporting tolerance to the mycotoxin. By 48 hpi, we observe a significant reduction of yeast cells producing the empty vector or ß-tubulin in the presence of 15-ADON compared to the absence of 15-ADON, whereas growth remains consistent for yeast cells producing UGT-6 or ubiquinol oxidases in the presence or absence of 15-DON (Fig. 5*D*). These data further suggest that production of UGT-6 or ubiquinol oxidase promotes yeast cell tolerance to 15-DON as demonstrated through growth. To tease apart if yeast cell survival at 24 hpi was attributed to tolerance or growth, we performed complementary CFU counts and we observed a similar trend with elevated recovery of yeast cells upon production of ubiquinol oxidase at 24 hpi, suggesting an ability of the yeast to not only tolerate but also proliferate in the presence of 15-DON (Fig. 5*E*). Taken together, these data support the identification and characterization of novel DON detoxifying proteins within the wheat proteome. Our next studies focus upon teasing apart the mechanisms of tolerance, survival, detoxification, and growth by exploring metabolic breakdown products of DON and additional prioritized candidates with putative roles in DON detoxification, including other oxidases.

## Discussion

The annual yield of wheat (*T. aestivum*) and other cereal crops globally can be heavily influenced by infection with *F. graminearum* and its corresponding disease FHB, as well as the production of the mycotoxin, DON. While resistance to FHB and DON are present within specific cultivars of *T. aestivum* (i.e., Sumai#3), more research is needed to further define the biochemical mechanisms involved in FHB resistance. Known methods by which *T. aestivum* detoxifies DON include utilizing glycosyltransferases and glutathione transferases to add functional groups to the mycotoxin, which signals for movement of the mycotoxin to the vacuole as a protective mechanism for the plant (36). In this study, we defined the cultivar-specific responses of wheat to 15-ADON exposure at the protein level. We assessed the impact of diverse 15-ADON levels (i.e., 0.1 mg/ml and 1.0 mg/ml) to mimic conditions within a field during years of moderate to severe infection and across a continuum of infection (i.e., 24 and 120 hpi) to define stages of the plant’s defenses to 15-ADON exposure from immediate to prolonged. On a global scale, we observed distinction across the tested conditions based on time and treatment as well as a substantial core proteome shared amongst the samples. We explored cultivar-specific responses to 15-ADON and observed a common suppression of photosynthesis in the presence of the mycotoxin, along with a reduction in defense responses within the FHB-susceptible cultivar. Relevant to DON detoxification, we observed a significant increase in the production of glycosyltransferases and glutathione-associated proteins within the FHB-resistant cultivar, supporting a specific protective mechanism. Further investigation into such protection against DON identified proteins universally detected across cultivars, time, and 15-ADON concentrations, and determined a role for oxidases in DON tolerance.

To explore the relevance of the selected ubiquinol oxidase and propose mechanistic insights into the displayed DON detoxification, we assessed its sequence homology. Herein, we observed a 99.3% shared identity with a wheat alternative oxidase, known as TaAOX1, which was previously associated with the *Fhb1* QTL in cultivar CMB82036 to enhance the DON-tolerance (61). AOX1, also known as mitochondrial alternative oxidase, located at the end of the plant mitochondrial respiratory pathway, was suggested to respond to ROS formation. Previously, when the homologous gene of *Fhb1-associated* AOX was overexpressed in *Arabidopsis*, abiotic oxidative stress was alleviated (63). Specifically, as DON triggers host programmed cell death through an influx of ROS, when Sumai#3 was inoculated with 3- and 15-ADON, oxidases responded in a race-specific response to the 15-ADON chemotype infection (26, 64). Thus, we selected the ubiquinol oxidase, which is located on chromosome 2A (assessed through the International Wheat Genome Sequencing Consortium), to evaluate the putative ROS-induced cellular damage caused by 15-ADON. An *in vitro* growth assay using a yeast expression system showed that yeast survival upon induction of a ubiquinol oxidase in the presence of 15-ADON provided greater protective capacity than the positive control with well-defined roles in DON detoxification. Overall, our study provides the first in-depth proteome profiling of 15-ADON exposure across wheat cultivars and proposes new strategies for enhanced elimination of DON from grains to promote increased food safety and security.

Previous transcriptome studies in wheat and barley support the role of drug efflux systems, secondary metabolite transformation and detoxification, and oxidative stress response as important components of the host response to DON (58, 61, 65, 66, 67, 68). Our findings at the protein level align with these observations and confirm dynamic proteome responses of the plants in response to DON. For instance, chitinases are PR proteins that plants use to combat infection by fungi through the degradation of the pathogenic cell wall (24, 25, 69). In our study, we observed an immediate decrease in chitinase production by both Norwell and Sumai#3 inoculated with the high 15-ADON concentration, suggesting a basal response to the presence of elevated DON toward chitinase inhibition. These inhibitory effects may be driven by the mycotoxin, or the host itself. Additional transcriptomic profiling under a range of DON concentrations and time did not show an increase in chitinase transcript expression in the presence of DON, supporting a degradative role of mycotoxins to evade this host defense response (58). Within our study, we also observed the importance of general plant defense responses upon DON exposure. For instance, the lack of a significant decrease in defense response proteins among the FHB-resistant cultivar suggests that Sumai#3 may continuously produce proteins to aid in survival after long exposure to the mycotoxin, whereas the FHB-susceptible cultivar, Norwell, either begins to lose this ability or shifts energy away from defense response protein production in the presence of DON. Given our observation of reduced protein folding and translation in Norwell at an earlier time point, under response to both low and high 15-ADON concentrations, and the known role of DON towards inhibition of protein synthesis (70), our findings suggest an inability of the FHB-susceptible cultivar to maintain general defense response proteins under stress.

For protection against DON through detoxification, wheat activates glycosyltransferases to add glucose molecules to DON at either the C3 or C15 hydroxyl groups, reducing the ribosomal binding potential for the mycotoxin by increasing the size of the molecule (30, 34, 36). Additionally, the glycosylated DON conjugate is shuttled to the vacuole for storage and/or potential further degradation reactions (31, 71). The increase in glycosyltransferase production as found in the FHB-resistant Sumai#3 cultivar, provides insightful evidence that such proteins are involved in host tolerance to DON. Currently, there is no known detoxication of the glycosylated DON conjugate beyond its allocation to the vacuole, and as such, it is likely that the plant uses glycosylation to detoxify and store the toxin. While D3G is often characterized as a masked mycotoxin due to its ability to release DON after hydrolytic processes, glycosyltransferases may be useful in reducing the ability of *F. graminearum* to spread through the host, and as a result, decreasing the overall presence of the mycotoxin throughout the plant system. Notably, we did not identify these “classical” DON detoxification signatures within our prioritized list of candidates associated with DON detoxification. This is likely due to differential production of glycosyltransferases across the cultivars, time points, and DON concentrations, indicating that these proteins are not universally activated, but rather provide an advantageous mechanism for DON detoxification. For the *in vitro* DON tolerance assay, we selected UGT6 as a positive control and evaluated the potential of ubiquinol oxidase in promoting DON tolerance within the system. Our findings suggest that the oxidase is more effective than UGT6 in promoting yeast growth in the presence of DON, suggesting a novel avenue for DON detoxification. However, we present several limitations and require further exploration *in planta* along with quantification of DON and putative detoxification targets within the yeast growth media to propose a novel mechanism for detoxification. Exploration of these proteins as future breeding targets and their effects on total DON production within infected plants could further support this hypothesis.

Another notable method used by *T. aestivum* in DON detoxication is the production of GSTs (57). The addition of GSH to xenobiotics and mycotoxins, such as 15-ADON, results in reduced toxicity, as well as the movement of the conjugate to the vacuole (35). We focused on the abundance of known GSTs and GST-domain containing proteins upon treatment with 15-ADON. The significant increase in these proteins within the Sumai#3 samples treated with 15-ADON and a lack of difference in the Norwell samples, supports a connection between DON detoxification and FHB resistance. Moreover, the increase in these proteins under both low and high concentrations of 15-ADON highlights their importance in general response for Sumai#3. The exploration of GSTs as lead breeding targets may be a valuable approach as the addition of GSH at the epoxide would support resistance across all known chemotypes of DON, as well as other trichothecenes with the same epoxide groups (28, 72). Again, we did not identify GST or GST domain-containing proteins within the list of prioritized DON detoxifying proteins, indicating the role of cultivar, time, and DON concentration-dependent responses that provide beneficial properties for the FHB-resistant cultivar to tolerate dangerous mycotoxins.

## Conclusion

Overall, our study complements previous phenotypic and transcriptomic studies by providing new insights in mycotoxin tolerance and detoxification at the protein level. Our findings highlight dynamic regulatory mechanisms activated upon DON exposure that may also play important roles in protection against FHB. Moreover, we confirm the production of known DON detoxification proteins, and we demonstrate a proof-of-principle study for the identification of novel detoxification strategies independent of wheat cultivar, suggesting universal mechanisms. These findings warrant further exploration via *in planta* testing to assess cultivar-specific DON detoxification strategies to drive the discovery of new biomarkers for selected breeding of wheat varieties with improved tolerance and protective mechanisms toward DON.

## Data Availability

The proteomics datasets are publicly available through PRIDE Proteomics Exchange: PXD059826.

## Supplemental Data

This article contains supplemental data.

## Conflict of Interest

The authors declare that they have no conflicts of interest with the contents of this article.

## References

[1] Awika J.M. (2011).

[2] Loskutov I.G. (2021). Advances in cereal crops breeding. Plants.

[3] Figueroa M., Hammond-Kosack K.E., Solomon P.S. (2018). A review of wheat diseases—a field perspective. Mol. Plant Pathol..

[4] Wilson W., Dahl B., Nganje W. (2018). Economic costs of Fusarium Head Blight, scab and deoxynivalenol. World Mycotoxin J..

[5] Liu B., Stevens-Green R., Johal D., Buchanan R., Geddes-McAlister J. (2022). Fungal pathogens of cereal crops: proteomic insights into fungal pathogenesis, host defense, and resistance. J. Plant Physiol..

[6] Janaviciene S., Suproniene S., Kadziene G., Pavlenko R., Berzina Z., Bartkevics V. (2022). Toxigenicity of *F. graminearum* residing on host plants alternative to wheat as influenced by environmental conditions. Toxins (Basel).

[7] Brauer E.K., Subramaniam R., Harris L.J. (2020). Regulation and dynamics of gene expression during the life cycle of *Fusarium graminearum*. Phytopathology.

[8] Audenaert K., Vanheule A., Höfte M., Haesaert G. (2013). Deoxynivalenol: a major player in the multifaceted response of *Fusarium* to its environment. Toxins (Basel).

[9] De Almeida J.L., Tessmann D.J., Do Couto H.T.Z., Fostim M.L. (2016). Effect of Fusarium head blight on deoxynivalenol levels in whole grain and patent flours from different wheat genotypes. World Mycotoxin J..

[10] Foroud N.A., Baines D., Gagkaeva T.Y., Thakor N., Badea A., Steiner B. (2019). Trichothecenes in cereal grains – an update. Toxins (Basel).

[11] Lei Y., Guanghui Z., Xi W., Yingting W., Xialu L., Fangfang Y. (2017). Cellular responses to T-2 toxin and/or deoxynivalenol that induce cartilage damage are not specific to chondrocytes. Sci. Rep..

[12] Pierron A., Mimoun S., Murate L.S., Loiseau N., Lippi Y., Bracarense A.-P.F.L. (2016). Microbial biotransformation of DON: molecular basis for reduced toxicity. Sci. Rep..

[13] Villafana R.T., Ramdass A.C., Rampersad S.N. (2019). Selection of *Fusarium* trichothecene toxin genes for molecular detection depends on TRI gene cluster organization and gene function. Toxins (Basel).

[14] Rocha O., Ansari K., Doohan F.M. (2005). Effects of trichothecene mycotoxins on eukaryotic cells: a review. Food Addit. Contam..

[15] Mishra S., Dwivedi P.D., Pandey H.P., Das M. (2014). Role of oxidative stress in Deoxynivalenol induced toxicity. Food Chem. Toxicol..

[16] Jia L.-J., Tang H.-Y., Wang W.-Q., Yuan T.-L., Wei W.-Q., Pang B. (2019). A linear nonribosomal octapeptide from *Fusarium graminearum* facilitates cell-to-cell invasion of wheat. Nat. Commun..

[17] Pinton P., Oswald I. (2014). Effect of deoxynivalenol and other type B trichothecenes on the intestine: a review. Toxins (Basel).

[18] Holanda D.M., Yiannikouris A., Kim S.W. (2020). Investigation of the efficacy of a postbiotic yeast cell wall-based blend on newly-weaned pigs under a dietary challenge of multiple mycotoxins with emphasis on deoxynivalenol. Toxins (Basel).

[19] Perlikowski D., Wiśniewska H., Góral T., Kwiatek M., Majka M., Kosmala A. (2014). Identification of kernel proteins associated with the resistance to Fusarium head blight in winter wheat (*Triticum aestivum* L.). PLoS One.

[20] Kage U., Hukkeri S., Kushalappa A.C. (2017). Liquid chromatography and high-resolution mass spectrometry-based metabolomics to identify quantitative resistance-related metabolites and genes in wheat QTL-2DL against Fusarium head blight. Eur. J. Plant Pathol..

[21] Gunupuru L.R., Perochon A., Doohan F.M. (2017). Deoxynivalenol resistance as a component of FHB resistance. Trop. Plant Pathol..

[22] Schweiger W., Steiner B., Ametz C., Siegwart G., Wiesenberger G., Berthiller F. (2013). Transcriptomic characterization of two major *Fusarium* resistance quantitative trait loci (QTLs), *Fhb1* and *Qfhs.ifa-5A*, identifies novel candidate genes. Mol. Plant Pathol..

[23] Lemmens M., Scholz U., Berthiller F., Dall’Asta C., Koutnik A., Schuhmacher R. (2005). The ability to detoxify the mycotoxin deoxynivalenol colocalizes with a major quantitative trait locus for Fusarium head blight resistance in wheat. Mol. Plant-Microbe Interact..

[24] Caruso C., Caporale C., Chilosi G., Vacca F., Bertini L., Magro P. (1996). Structural and antifungal properties of a pathogenesis-related protein from wheat kernel. J. Protein Chem..

[25] Caporale C., Di Berardino I., Leonardi L., Bertini L., Cascone A., Buonocore V. (2004). Wheat pathogenesis-related proteins of class 4 have ribonuclease activity. FEBS Lett..

[26] Desmond O.J., Manners J.M., Stephens A.E., Maclean D.J., Schenk P.M., Gardiner D.M. (2008). The *Fusarium* mycotoxin deoxynivalenol elicits hydrogen peroxide production, programmed cell death and defence responses in wheat. Mol. Plant Pathol..

[27] Ding L., Xu H., Yi H., Yang L., Kong Z., Zhang L. (2011). Resistance to hemi-biotrophic *F. graminearum* infection is associated with coordinated and ordered expression of diverse defense signaling pathways. PLoS One.

[28] Wang H., Sun S., Ge W., Zhao L., Hou B., Wang K. (2020). Horizontal gene transfer of *Fhb7* from fungus underlies Fusarium head blight resistance in wheat. Science.

[29] Gunnaiah R., Kushalappa A.C., Duggavathi R., Fox S., Somers D.J. (2012). Integrated metabolo-proteomic approach to decipher the mechanisms by which wheat QTL (Fhb1) contributes to resistance against *Fusarium graminearum*. PLoS One.

[30] He Y., Ahmad D., Zhang X., Zhang Y., Wu L., Jiang P. (2018). Genome-wide analysis of family-1 UDP glycosyltransferases (UGT) and identification of UGT genes for FHB resistance in wheat (*Triticum aestivum* L.). BMC Plant Biol..

[31] Schmeitzl C., Warth B., Fruhmann P., Michlmayr H., Malachová A., Berthiller F. (2015). The metabolic fate of deoxynivalenol and its acetylated derivatives in a wheat suspension culture: identification and detection of DON-15-O-glucoside, 15-acetyl-DON-3-O-glucoside and 15-acetyl-DON-3-sulfate. Toxins (Basel).

[32] Poppenberger B., Berthiller F., Lucyshyn D., Sieberer T., Schuhmacher R., Krska R. (2003). Detoxification of the *Fusarium* mycotoxin deoxynivalenol by a UDP-glucosyltransferase from *Arabidopsis thaliana*. J. Biol. Chem..

[33] He Y., Wu L., Liu X., Jiang P., Yu L., Qiu J. (2020). TaUGT6, a novel UDP-glycosyltransferase gene enhances the resistance to FHB and DON accumulation in wheat. Front. Plant Sci..

[34] Uhlig S., Stanic A., Hofgaard I., Kluger B., Schuhmacher R., Miles C. (2016). Glutathione-conjugates of deoxynivalenol in naturally contaminated grain are primarily linked via the epoxide group. Toxins (Basel).

[35] Kluger B., Bueschl C., Lemmens M., Michlmayr H., Malachova A., Koutnik A. (2015). Biotransformation of the mycotoxin deoxynivalenol in *Fusarium* resistant and susceptible near isogenic wheat lines. PLoS One.

[36] Berthiller F., Crews C., Dall’Asta C., Saeger S. De, Haesaert G., Karlovsky P. (2013). Masked mycotoxins: a review. Mol. Nutr. Food Res..

[37] Geddes J., Eudes F., Laroche A., Selinger L.B. (2008). Differential expression of proteins in response to the interaction between the pathogen *Fusarium graminearum* and its host, hordeum vulgare. Proteomics.

[38] Zhao Y., Zhang L., Ju C., Zhang X., Huang J. (2022). Quantitative multiplexed proteomics analysis reveals reshaping of the lysine 2-hydroxyisobutyrylome in *Fusarium graminearum* by tebuconazole. BMC Genomics.

[39] Willforss J., Leonova S., Tillander J., Andreasson E., Marttila S., Olsson O. (2020). Interactive proteogenomic exploration of response to Fusarium head blight in oat varieties with different resistance. J. Proteomics.

[40] Yang M., Wang X., Dong J., Zhao W., Alam T., Thomashow L.S. (2021). Proteomics reveals the changes that contribute to Fusarium head blight resistance in wheat. Phytopathology.

[41] Fabre F., Urbach S., Roche S., Langin T., Bonhomme L. (2021). Proteomics-based data integration of wheat cultivars facing *Fusarium graminearum* strains revealed a core-responsive pattern controlling Fusarium head blight. Front. Plant Sci..

[42] Sobrova P., Adam V., Vasatkova A., Beklova M., Zeman L., Kizek R. (2010). Deoxynivalenol and its toxicity. Interdiscip. Toxicol..

[43] Ngundi M.M., Qadri S.A., Wallace E.V., Moore M.H., Lassman M.E., Shriver-Lake L.C. (2006). Detection of deoxynivalenol in foods and indoor air using an array biosensor. Environ. Sci. Technol..

[44] Zhai Y., Hu S., Zhong L., Lu Z., Bie X., Zhao H. (2019). Characterization of deoxynivalenol detoxification by *Lactobacillus paracasei* LHZ-1 isolated from yogurt. J. Food Prot..

[45] Buchanan R., Serajazari M., Geddes-McAlister J., Foroud N.A., Neilson J.A.D. (2023). Plant-Pathogen Interactions. Methods in Molecular Biology.

[46] Wiśniewski J.R., Gaugaz F.Z. (2015). Fast and sensitive total protein and Peptide assays for proteomic analysis. Anal. Chem..

[47] Rappsilber J., Mann M., Ishihama Y. (2007). Protocol for micro-purification, enrichment, pre-fractionation and storage of peptides for proteomics using StageTips. Nat. Protoc..

[48] Thompson A., Schäfer J., Kuhn K., Kienle S., Schwarz J., Schmidt G. (2003). Tandem mass tags: a novel quantification strategy for comparative analysis of complex protein mixtures by MS/MS. Anal. Chem..

[49] Cox J., Mann M. (2008). MaxQuant enables high peptide identification rates, individualized p.p.b.-range mass accuracies and proteome-wide protein quantification. Nat. Biotechnol..

[50] Cox J., Neuhauser N., Michalski A., Scheltema R.A., Olsen J.V., Mann M. (2011). Andromeda: a peptide search engine integrated into the MaxQuant environment. J. Proteome Res..

[51] (2021). UniProt: the universal protein knowledgebase in 2021. Nucleic Acids Res..

[52] Tyanova S., Temu T., Sinitcyn P., Carlson A., Hein M.Y., Geiger T. (2016). The Perseus computational platform for comprehensive analysis of (prote) omics data. Nat. Methods.

[53] Olabisi-Adeniyi E., McAlister J.A., Ferretti D., Cox J. (2025). ProteoPlotter: an executable proteomics visualization tool compatible with Perseus. J. Proteome Res..

[54] Ebeling J., Fünfhaus A., Knispel H., Krska D., Ravulapalli R., Heney K.A. (2017). Characterization of the toxin Plx2A, a RhoA-targeting ADP-ribosyltransferase produced by the honey bee pathogen *Paenibacillus larvae*. Environ. Microbiol..

[55] Benatuil L., Perez J.M., Belk J., Hsieh C.-M. (2010). An improved yeast transformation method for the generation of very large human antibody libraries. Protein Eng. Des. Selection.

[56] Cox J., Mann M. (2012). 1D and 2D annotation enrichment: a statistical method integrating quantitative proteomics with complementary high-throughput data. BMC Bioinformatics.

[57] Govindarajan S., Mannervik B., Silverman J.A., Wright K., Regitsky D., Hegazy U. (2015). Mapping of amino acid substitutions conferring herbicide resistance in wheat glutathione transferase. ACS Synth. Biol..

[58] Walter S., Doohan F. (2011). Transcript profiling of the phytotoxic response of wheat to the Fusarium mycotoxin deoxynivalenol. Mycotoxin Res..

[59] Nishiuchi T., Masuda D., Nakashita H., Ichimura K., Shinozaki K., Yoshida S. (2006). *Fusarium* phytotoxin trichothecenes have an elicitor-like activity in *Arabidopsis thaliana*, but the activity differed significantly among their molecular species. Mol. Plant-Microbe Interactions®.

[60] Park J., Lee H.-H., Youn K., Kim S., Jung B., Lee J. (2014). Transcriptome analyses to understand effects of the Fusarium deoxynivalenol and nivalenol mycotoxins on *Escherichia coli*. J. Biotechnol..

[61] Walter S., Brennan J.M., Arunachalam C., Ansari K.I., Hu X., Khan M.R. (2008). Components of the gene network associated with genotype-dependent response of wheat to the *Fusarium* mycotoxin deoxynivalenol. Funct. Integr. Genomics.

[62] Lyagin I., Maslova O., Stepanov N., Senko O., Efremenko E. (2025). Reassessing of enzymes degrading mycotoxins at acidic pH. Int. Biodeterior Biodegradation.

[63] Sugie A., Naydenov N., Mizuno N., Nakamura C., Takumi S. (2006). Overexpression of wheat alternative oxidase gene Waox1a alters respiration capacity and response to reactive oxygen species under low temperature in transgenic Arabidopsis. Genes Genet. Syst..

[64] Al-Taweel K., Fernando W.G.D., Brûlé-Babel A.L. (2014). Transcriptome profiling of wheat differentially expressed genes exposed to different chemotypes of *Fusarium graminearum*. Theor. Appl. Genet..

[65] Boddu J., Cho S., Muehlbauer G.J. (2007). Transcriptome analysis of trichothecene-induced gene expression in barley. Mol. Plant-Microbe Interactions®.

[66] Gardiner S.A., Boddu J., Berthiller F., Hametner C., Stupar R.M., Adam G. (2010). Transcriptome analysis of the barley–deoxynivalenol interaction: evidence for a role of glutathione in deoxynivalenol detoxification. Mol. Plant-Microbe Interactions®.

[67] Doohan F., Arunachalam C., Jiang S., Khan M., Egan D., Erard G. (2008). The wheat response to deoxynivalenol: does maintenance of hormone homeostasis and alleviation of oxidative stress play an important role in toxin tolerance?. Cereal Res. Commun..

[68] Ansari K.I., Walter S., Brennan J.M., Lemmens M., Kessans S., McGahern A. (2007). Retrotransposon and gene activation in wheat in response to mycotoxigenic and non-mycotoxigenic-associated *Fusarium* stress. Theor. Appl. Genet..

[69] Franco F.P., Dias R.O., Toyama D., Henrique-Silva F., Moura D.S., Silva-Filho M.C. (2019). Structural and functional characterization of PR-4 SUGARWINs from sugarcane and their role in plant defense. Front. Plant Sci..

[70] Miller J.D., Ewen M.A. (1997). Toxic effects of deoxynivalenol on ribosomes and tissues of the spring wheat cultivars Frontana and Casavant. Nat. Toxins.

[71] Ovando-Martínez M., Ozsisli B., Anderson J., Whitney K., Ohm J.-B., Simsek S. (2013). Analysis of deoxynivalenol and deoxynivalenol-3-glucoside in hard red spring wheat inoculated with *Fusarium graminearum*. Toxins (Basel).

[72] McCormick S.P., Stanley A.M., Stover N.A., Alexander N.J. (2011). Trichothecenes: from simple to complex mycotoxins. Toxins (Basel).

