# Supplementary material for: Temporal Wheat Proteome Remodeling by Deoxynivalenol Reveals Novel Detoxification Signatures and Strategies Across Cultivars
Source: Mol Cell Proteomics. 2025 May 9;24(6):100988. doi: 10.1016/j.mcpro.2025.100988 (PMC12221369; doi:10.1016/j.mcpro.2025.100988)
Supplement: Supplemental Figure S1 [file mmc1.docx]

**
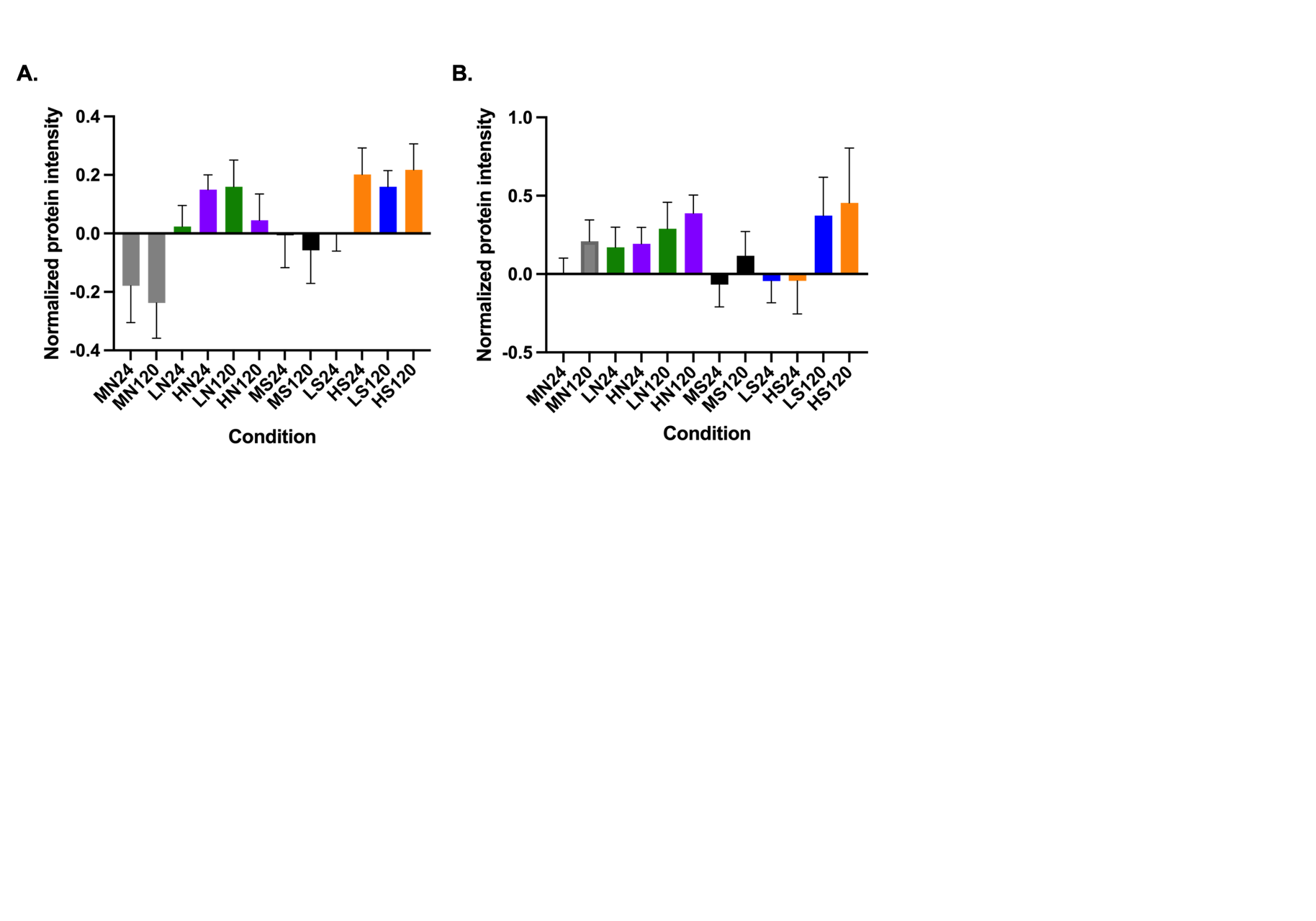
**

**Figure S1.** **Comparison of normalized intensities for proteins associated with programmed cell death. A.** Uncharacterized protein, A0A3B6NNF5. **B.** MAP kinase protein, A0A3B5ZTL2. Bar plot with standard error bars; 10 biological replicates. MN = Mock Norwell, MS = Mock Sumai#3, LN = low DON Norwell, HN, high DON Norwell, LS, low DON Sumai#3, HS = high DON Sumai#3.
